# Characterization of a novel aminoglycoside resistance gene, *aadA34*, identified from *Serratia ureilytica* S24

**DOI:** 10.3389/fmicb.2026.1765554

**Published:** 2026-03-18

**Authors:** Chaoqun Liu, Zhigang Yang, Shenghai Wu, Huiyao Cao, Qing Wenren, Yanfang Zhang, Huiyue Feng, Junwan Lu, Xiaojun Xu, Xiaowei Chen, Qiyu Bao, Teng Xu, Wei Lu

**Affiliations:** 1 Medical Molecular Biology Laboratory, School of Medicine, Jinhua University of Vocational Technology, Jinhua, China; 2 Institute of Translational Medicine, Baotou Central Hospital, Baotou, China; 3 Department of Laboratory Medicine, Hangzhou First People’s Hospital Affiliated of Westlake University School of Medicine, Hangzhou, China; 4 Zhejiang Pharmaceutical University, Ningbo, China; 5 Institute of Biomedical Informatics/School of Laboratory Medicine and Life Sciences, Wenzhou Medical University, Wenzhou, China

**Keywords:** *aadA34*, aminoglycoside nucleotidyltransferase, antimicrobial resistance gene, kinetic parameter, *Serratia ureilytica*

## Abstract

Bacterial resistance to aminoglycoside drugs is becoming increasingly severe due to their widespread use in agriculture and human medicine. Based on the whole-genome sequencing and molecular methods, a novel aminoglycoside resistance gene designated *aadA34* was identified from a multiple-drug-resistant bacterium *Serratia ureilytica* S24, which was isolated from sewage at a poultry farm. The *aadA34* gene conferred resistance to spectinomycin and streptomycin, with MIC levels increased by 64- and 128-fold, respectively, for spectinomycin and streptomycin compared with the control strain (pUCP20/*E. coli* DH5α). The amino acid (aa) sequence of AadA34 was found to share the highest identity (60.09%) with the function-characterized aminoglycoside nucleotidyltransferase AadA16. Corresponding to the MIC results of the *aadA34* gene, the enzyme AadA34 exhibited high affinity and catalytic efficiency against spectinomycin or streptomycin. Approximately 160 aa longer than other functionally characterized AadA proteins, the *aadA34* gene was found to be generally encoded in bacteria of the genus *Serratia*, especially in the species *S. ureilytica* and *S. marcescens*, which were isolated from various sources including some of clinical significance.

## Highlights

The rise of bacterial resistance to aminoglycosides, spurred by their extensive use in agriculture and medicine, poses a threat to the effective treatment of infections. The discovery of a novel chromosomally encoded aminoglycoside nucleotidyltransferase gene, *aadA34*, from an environmental bacterium, *S. ureilytica* S24, which is conserved across different species of the genus *Serratia* from diverse sources, including some of clinical importance, will assist in the treatment of animal and human infectious diseases caused by related bacterial species.

## Introduction

The emergence of antimicrobial resistance in common clinical pathogens has made it more difficult to manage infectious diseases based on conventional treatment strategies in humans as well as in animals. Bacterial resistance is the most serious threat to public health and causes considerable mortality and morbidity.

As has been extensively demonstrated, the abuse of antimicrobial drugs in agriculture and husbandry can increase the prevalence of drug-resistant bacteria (Butaye et al., 2014; Heuer et al., 2009; Manaia, 2017). An even more serious issue is that gram-negative bacteria become resistant to many advanced antibiotics, such as carbapenems and polymyxins, exacerbating the process of returning to the preantibiotic era (Nordmann and Poirel, 2019; Rhouma et al., 2016). Based on these situations, the use of some “old” antibiotics, such as aminoglycosides, fosfomycin, and chloramphenicol, is now being revived in hospitals and other fields, as they are known to have strong antibacterial effects on multidrug-resistant organisms (Theuretzbacher et al., 2015). Notably, aminoglycoside antibiotics have been widely used as bactericidal drugs in both veterinary and clinical settings for many years. The first aminoglycoside, streptomycin, discovered by Waksman and successfully used to treat infectious diseases, is still used today (Schatz et al., 2005). The specific structure of the aminocyclitol antibiotic spectinomycin was subsequently identified from *Streptomyces spectabilis*. Spectinomycin has been shown to be effective for treating gonorrhea infection (Holloway, 1982). However, in the era of antibiotic abuse, a large number of aminoglycoside resistance genes might not yet be identified in many microorganisms.

Aminoglycoside-modifying enzymes, as the most significant and most popular drug resistance mechanism in pathogens, catalyze the modification of the –NH_2_ or –OH groups of the 2-deoxystreptamine nucleus and can be designed as acetyltransferases (AACs), adenylyltransferases (Aads) or phosphotransferases (APHs). The first *aadA* gene was discovered in Tn*1331* (Tolmasky, 1990), which is specifically resistant to streptomycin and spectinomycin. The *aadA* gene is thought to have originated from environmental bacteria. Aminoglycosides are widely used in agriculture as growth promoters and have been applied in veterinary medicine. This has led to the selection of resistance genes in the environment, which can then be transferred to human pathogens. This emergence mechanism is supported by the fact that the *aadA* gene has been found in environmental isolates such as *Pseudomonas* and *Aeromonas* (Nguyen et al., 2014). Such genes have been widely found in clinically common microorganisms, such as *Pseudomonas aeruginosa*, *Klebsiella pneumoniae*, and *Acinetobacter baumannii*. Several studies on food-derived pathogens, such as *Salmonella* and *S. maltophilia*, have proven that the extensive application of antibiotics in husbandry can cause refractory infections in patients (Brooke, 2012; Mukherjee et al., 2019). Although numerous studies have focused on the exploration of the prevalence and pathogenic mechanisms of bacteria from veterinary and food resources, few studies have investigated aminoglycoside-resistant organisms isolated from livestock and food products, and even fewer have investigated environmental bacteria. Hence, understanding the genetic characterization and origination of a novel resistance mechanism from various sources is important in the development of strategies to combat antibiotic resistance.

*Serratia* species are gram-negative bacteria that widely exist in environments around husbandry farms and hospitals (Mahlen, 2011). Several studies have demonstrated that *Serratia* spp. include important pathogens that have caused nosocomial outbreaks (Dahdouh et al., 2021). To date, a great number of *Serratia* spp. harboring resistance determinants encoded on chromosomes or plasmids have been reported, including some carbapenemases that have been associated with *Serratia* outbreaks (Hopkins et al., 2017; Messaoudi et al., 2021). Studies investigating aminoglycoside resistance in *E. coli* and many common microbes have been carried out, but the mechanism and prevalence of aminoglycoside resistance in *Serratia* spp. are still unclear. In this study, we aimed to characterize a novel streptomycin/spectinomycin adenylyltransferase gene, designated *aadA34*, based on molecular cloning, sequencing and enzyme kinetic analyses.

## Materials and methods

### Bacterial strains, plasmids and culture conditions

The isolate *Serratia ureilytica* S24 was isolated in July 2019 from sewage at a poultry farm in Wenzhou, China. Species identification was initially performed using a Vitek-60 microorganism autoanalysis system (BioMerieux Corporate, Craponne, France). Further identification was conducted by combining 16S rRNA homology and ANI identity analyses. *E. coli* DH5α was used as the host for cloning the resistance gene, and *E. coli* BL21(DE3) was used as the host for expression of the AadA34 enzyme. The plasmid pUCP20 (Takara, Dalian, China) was used as the vector for cloning resistance determinants. pCold I (OBiO Technology, Shanghai, China) was used for cold shock-induced expression and purification of histidine-tagged AadA34. The bacterial strains were cultured overnight in Luria–Bertani (LB) medium at 37 °C, supplemented with the corresponding antimicrobial agents and/or solidified with 1.5% agar when necessary, unless otherwise noted (Li et al., 2018) (Supplementary Table S1).

### Whole-genome sequencing and bioinformatics analysis

The genomic DNA of *Serratia ureilytica* S24 was extracted using an AxyPrep Bacterial Genomic DNA Miniprep Kit (Qiagen, Union City, CA, United States). The sequence data were generated on the Illumina HiSeq-2500 and PacBio RS II platforms by Shanghai Personal Biotechnology Co., Ltd. (Shanghai, China). The sequence data quality was checked using FastQC v0.11.5 and Canu v1.8 software (Wingett and Andrews, 2018). PacBio long reads of approximately 10–20 kb were assembled. Through the mapping of Illumina sequencing reads onto the initial assembly, the potential misidentified bases were corrected using the Burrows–Wheeler Alignment tool (BWA) (Jo, 2016) and Genome Analysis Toolkit (GATK) (McKenna et al., 2010). Prokka v1.14.0 (Seemann, 2014) was used to predict ORFs, after which DIAMOND (Lawton et al., 2025) was used to annotate the functions of the predicted proteins against the UniProtKB/Swiss-Prot^1^ and NCBI nonredundant protein databases. ResFinder (Florensa et al., 2022) and Resistance Gene Identifier (RGI) (Alcock et al., 2023) software and the Comprehensive Antibiotic Resistance Database (CARD),^2^ were used to annotate resistance genes. Antimicrobial resistance genes were considered those ORFs whose similarities were ≥80.0% with the function-characterized resistance genes in the database. MEGA X software (Kumar et al., 2018) was used to perform multiple sequence alignment and to construct a neighbor-joining phylogenetic tree, and the phylogenetic tree was visualized using iTOL.^3^ Conserved motif analysis of the AadA34 sequence was performed using the MEME Suite.^4^ Genetic context diagrams were generated with version 2.2.2 of Easyfig (Sullivan et al., 2011). A circular map of a genome was constructed using the GCView Comparison Tool (Grin and Linke, 2011).

### Antibiotic susceptibility testing

In this study, the agar dilution method was used with Mueller–Hinton agar plates to evaluate the minimal inhibitory concentrations (MICs) of different antimicrobial agents (Wiegand et al., 2008). The MIC results were analyzed according to the Clinical and Laboratory Standard Institute (CLSI 2021), the European Committee on Antimicrobial Susceptibility Testing (ECAST) (version 11.0, 2021) and the National Antimicrobial Resistance Monitoring System (NARMS) for enteric bacteria breakpoint criteria (Franklin et al., 2024), and the criteria used to establish antibiotic breakpoints for *Serratia ureilytica* were based on those previously reported for *Enterobacter* spp. (Mischnik et al., 2017). *Escherichia coli* ATCC 25922 and *Pseudomonas aeruginosa* ATCC 27853 were included in each test as the quality control strains. All experiments were performed three times.

### Cloning of resistance genes

The genomic DNA of *S. ureilytica* S24 was extracted using the method mentioned above. The DNA fragment carrying the putative resistance gene and its promoter region was amplified by PCR using the primers listed in Table 1. The PCR products were digested with their corresponding restriction endonucleases, followed by the ligation of *aadA34* with the cloning vector pUCP20. The recombinant plasmid was introduced into *E. coli* DH5α via electroporation. The transformants were selected on LB agar plates that contained 100 mg/L ampicillin, 40 mg/L Xgal and 0.5 mM IPTG. The size and sequence of the cloned inserts were confirmed by restriction enzyme digestion and Sanger sequencing.

**Table 1:** Primers used in this study.

Primers	Sequences (5′ to 3′)	Product size	Enzyme site	Purpose
aadA34-EcoRI-F	CGGAATTCTCGCACACCTTAATGTGGTTTCATT	1,359 bp	*Eco*RI *Sph*I	For cloning *aadA34* with its promter region
aadA34-SphI-R	CATGCATGCTTATTGCGATGCTGTTGTCGTCAATA			
aadA34-ORF-XhoI-EK-F	CCCTCGAGGATGATGATAAGGTGAAACGTAAGAGCCTGGAAGACG	1,284 bp	*Xho*I + EK *Eco*RI	For cloning the ORF of *aadA34*
aadA34-ORF-EcoRI-R	CGGAATTCTTATTGCGATGCTGTTGTCGTCAATAGAC			

### Expression and purification of the AadA34 protein

Recombinant protein expression and purification were performed as previously described (Lu et al., 2021). Briefly, *E. coli* BL21(DE3) cells containing recombinant plasmids (pCold I-*aadA34*) were incubated in 500 mL of LB broth supplemented with 100 μg/mL ampicillin at 37 °C until an absorbance of 0.6–0.8 (OD_600_) was reached. The culture was chilled to 16 °C, and AadA34 expression was induced by the addition of 1.3 mM isopropyl-*β*-D-thiogalactoside (IPTG), followed by continuous incubation for 18 h at 16 °C. Cells were harvested by centrifugation (5,000 × g, 10 min) at 4 °C, resuspended in lysis buffer (20 mM Tris–HCl, 150 mM NaCl, 3 mM β-mercaptoethanol, 0.5% Nonidet-P-40, pH 8.0), and lysed by sonication. After insoluble debris was removed by centrifugation (12,000 × g, 30 min) at 4 °C, the lysate supernatant was purified using affinity chromatography with Ni-nitrilotriacetic acid (Beyotime Biotechnology, Shanghai, China) as the first purification step, and the His6-tag was removed by thrombin cleavage at 25 °C for 3 h, followed by another purification step with an ultrafiltration purification column to remove the free His6-tag. The purified AadA34 protein was enriched using an ultrafiltration spin column (Millipore, Massachusetts, United States) with a cutoff of 10 kDa. The purity and size of AadA34 were analyzed using SDS–PAGE (12%), and the protein concentration was measured by both a BCA protein assay and a spectrophotometric method (Cortes-Rios et al., 2020).

### *In vitro* enzyme activity detection of the aminoglycoside adenylyltransferase AadA34

The kinetic parameters for monitoring the enzyme activity of AadA34 against a panel of aminoglycosides (spectinomycin, streptomycin, gentamicin and kanamycin) were determined following previously described method (Kim et al., 2006). Briefly, the activity of AadA34 was detected by coupling the enzymatic reaction to the reactions of phosphoglucomutase, UDP-glucose pyrophosphorylase, and glucose-6-phosphate dehydrogenase. The catalytic activity of AadA34 was determined by monitoring NADPH accumulation at 340 nm using a Synergy^™^ Neo2 Multi-Mode Microplate Reader (BioTek Instruments, Inc., United States). The experiments were performed in a 96-well plate at 37 °C, for which the reaction mixtures contained 10 mM MgCl_2_, 50 mM HEPES (pH 7.5), 0.2 mM glucose, 1,6-bisphosphate, 0.2 mM dithiothreitol, 0.2 mM UDP-glucose, 0.2 mM NADP, 20 units/mL glucose-6-phosphate dehydrogenase, 2 units/mL UDP-glucose pyrophosphorylase, 20 units/mL phosphoglucomutase, 1 mM ATP, 3.41 × 10^−8^ mM AadA34, and varying aminoglycoside concentrations (2.5–170 μM) in a final volume of 200 μL. The reaction was initiated by the addition of AadA34 (0.5 μM final concentration), and the UV absorbance was measured over 5 min at 35 °C. All kinetic experiments were performed in triplicate, and data analysis was performed using GraphPad 9 software (Mitteer and Greer, 2022).

### Nucleotide sequence accession number

The nucleotide sequence data reported in this study have been deposited in GenBank under accession numbers CP076651 for the chromosome and OQ862328 for the *aadA34* gene of *Serratia ureilytica* S24.

## Results and discussion

### Species classification and antimicrobial resistance profile of *Serratia marcescens* S24

In recent years, a program investigating the resistance status of local animal and environmental bacteria has been conducted. An isolate named S24 was obtained from the sewage from a poultry farm in Wenzhou, China in 2019. Species classification was carried out first by 16S rRNA gene homology and then by whole-genome average nucleotide identity (ANI) analyses. Based on the genome sequences available in the public database, the 16S rRNA gene of S24 has the highest homologous relationship with that of *Serratia ureilytica* (GenBank assembly accession: GCA_017309605.1), followed by that of *Serratia marcescens* (GenBank assembly accession: GCA_017654245.1), with identities of 98.1 and 92.8%, respectively. ANI analysis revealed that the genome sequence sharing the highest ANI value (99.07%) with that of S24 was from the type strain *Serratia ureilytica* CCUG:50595^T^ (accession number in the NCBI assembly database: GCA_013375155.1). These results indicated that S24 is most closely related to *Serratia ureilytica* and was thus designated *Serratia ureilytica* S24. Most microbiological practices involve the proper identification of isolated bacterial strains. The different biochemical tests employed are based on the fact that each type of bacteria, owing to their specific metabolic properties, respond differently and yield certain positive or negative results. Molecular microbiology methods have revolutionized the bacterial identification process; these methods are not only faster and more accurate but also precise and broadly used. The 16S rRNA gene has become an ideal and essential DNA/gene fragment for the identification of bacteria and for comparative and phylogenetic studies and classification (Clarridge, 2004). However, at present, ANI comparison analysis has become the gold standard for bacterial identification (Thompson et al., 2013). It compares the whole-genome sequence and with type strains in the databases. When the similarity reaches more than 95.0%, the bacterium is considered to be from the same species as the compared bacteria (Versmessen et al., 2024).

*S. ureilytica* S24 was resistant to numerous antimicrobials tested, including aminoglycosides (64 mg/L of tribamycin and 32 mg/L of streptomycin), aminocyclitol (64 mg/L of spectinomycin), beta-lactams (ampicillin >512 mg/L, 64 mg/L of ceftiofur, and 8 mg/L of aztreonam), fosfomycin (128 mg/L), fluoroquinolone (64 mg/L of ciprofloxacin), tetracyclines (32 mg/L of tetracycline), polymyxin (polymyxin B > 8 mg/L), and trimethoprim (16 mg/L) (Supplementary Table S2). These results were consistent with those of previous studies. High resistance rates to ceftriaxone (43.2%), piperacillin/tazobactam (57.8%), ceftazidime (55.6%), cefepime (36.3%), trimethoprim/sulfamethoxazole (53.3%), gentamicin (48.8%), ciprofloxacin (44.5%), and amikacin (15.6%) have been documented among *Serratia ureilytica* isolates (Gomi et al., 2022).

### Genome features and aminoglycoside-resistant genotypes of *Serratia ureilytica* S24

**Figure 1:**
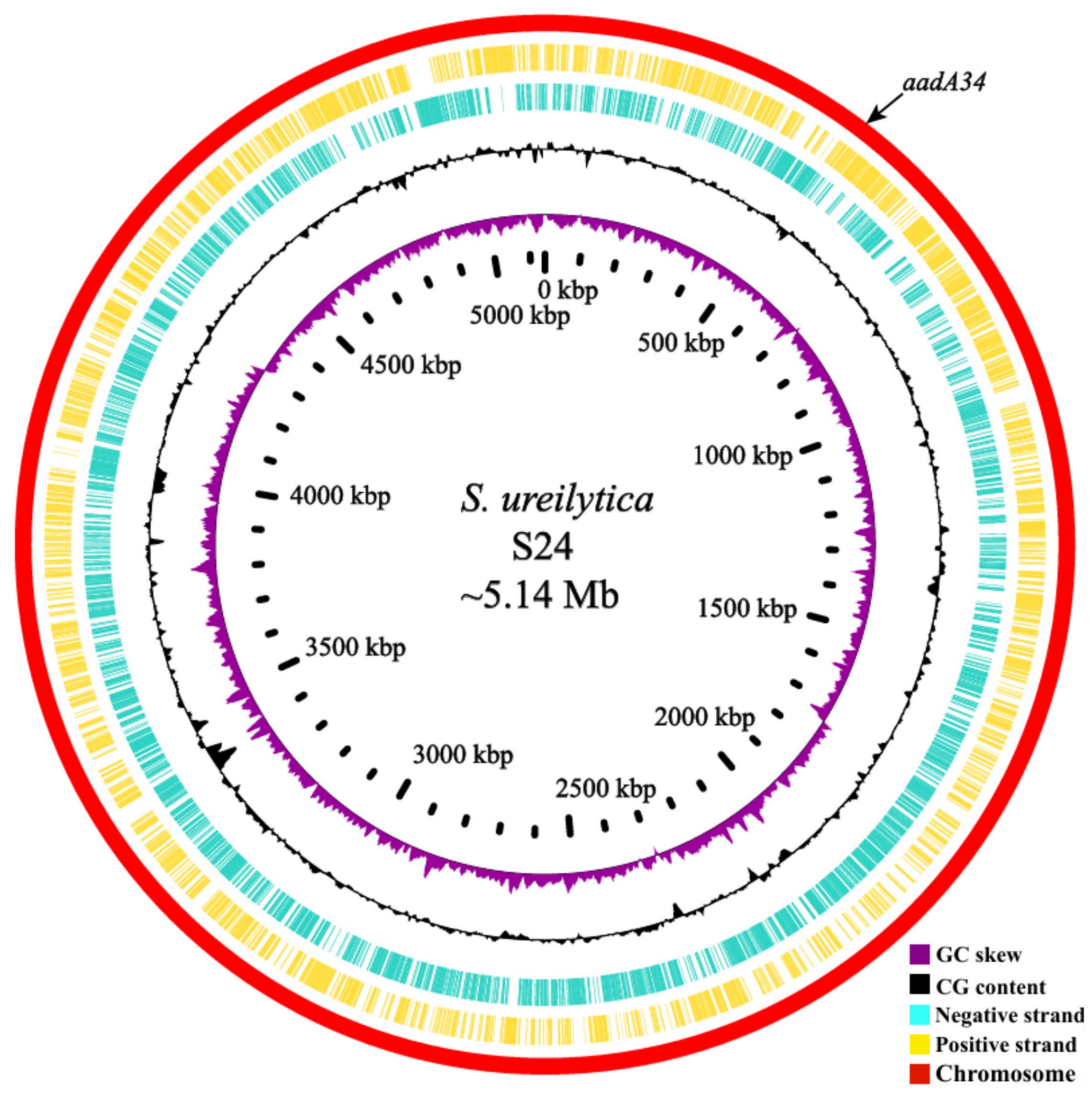
The genomic feature of *Serratia ureilytica* S24. Circular representation of chromosome of *S. ureilytica* S24 (GenBank accession number: CP076651). The black arrow indicates the chromosomal location of *aadA34*. From outside to inside: circle 1, representation of the *Serratia ureilytica* S24 genome; circles 2 and 3, predicted ORFs encoded in the plus and minus strands, respectively; circles 4 and 5, GC content and GC skew maps, respectively; circle 6, scale in kb (each tick is 100 kb).

To clarify the genetic basis of the multidrug resistance phenotype of S24, the whole genome of this strain was sequenced. It consisted of one circular chromosome that was 5.14 Mbp in length (GenBank accession number: CP076651), with an average GC content of 62.02% without a plasmid (Figure 1 and Table 2). The genome harbored a total of 4,773 protein-coding sequences, 22 rRNAs and 94 tRNA genes. Among the protein-coding sequences, only four were predicted to be resistance genes whose identities were ≥80% with the function-characterized resistance genes, including a beta-lactam (*bla*_SRT-2_), a tetracycline (*tet(41)*), a fluoroquinolone (*QnrB37*), and an aminoglycoside (*aac(6)-Ic*) resistance gene. The aminoglycoside resistance gene *aac(6)-Ic S. ureilytica* S24 generally confers resistance to gentamicin and amikacin (Shaw et al., 1992). In contrast, no genes conferring spectinomycin or/and streptomycin resistance were identified, even though S24 had high MICs of the two antimicrobial agents. These findings indicated that some unidentified resistance mechanisms might exist in the isolate. To investigate the resistance mechanism, six predicted ORFs (including *aadA-*, *aph(6)Ic-*, *aac(3)Ib-*, *aac(6′)Ib-*, *aac(6′)Iy-* and *aac(6′)Iak-*like genes) with <80.0% identities to functionally characterized aminoglycoside resistance genes were screened out from the annotation results of the whole-genome sequence. These six hypothetical aminoglycoside resistance genes were subsequently cloned, and their antimicrobial resistance functions were further verified (Supplementary Table S3).

**Table 2:** General genome features of *S. ureilytica* S24.

Characteristics	Chromosome
Size (bp)	5,142,018
GC content (%)	62
ORFs	4,773
Known proteins	4,743
Hypothetical proteins	30
Average ORF length (bp)	1,023
Average protein length (aa)	341
tRNAs	94
ncRNA	15
rRNAs	5S × 8; 16S × 7; 23S × 7

### *AadA34* is a novel aminoglycoside adenylyltransferase gene

Of the six genes cloned, only the *aadA-like* gene (ultimately designated *aadA34* in this work) was functional and showed distinctive resistance to streptomycin and spectinomycin. Compared with the control strain (pUCP20/*E. coli* DH5α), the recombinant harboring *aadA34* increased the MICs of spectinomycin and streptomycin by 64- and 128-fold, respectively, whereas the MICs of the other aminoglycosides, including kanamycin, ribostamycin, tobramycin, sisomicin, netilmicin, gentamicin, and amikacin, did not differ (Table 3). Ultimately, we named the novel resistance gene “*aadA34*” to follow the principles of numerical naming with the up-to-date resistance gene *aadA33* (Feng et al., 2022), avoiding confusion with the previously employed nomenclature.

**Table 3:** MICs of various aminoglycosides for *S. ureilytica* S24, the recombinants and the controls (μg/mL).

Bacterium	KAN	NEO	PAR	RIB	TOB	SIS	NET	MCR	APR	GEN	AMK	SPE	STR
ATCC25922	4	4	2	4	0.5	0.5	0.5	0.5	1	0.25	2	8	4
*S. ureilytica* S24	2	4	4	8	0.25	0.5	2	0.25	2	0.25	1	64	32
DH5α	1	2	2	2	0.25	0.25	0.25	0.25	1	0.25	1	8	2
DH5α/pUCP20	2	2	2	2	0.25	0.25	0.25	0.25	2	0.25	1	8	2
DH5α/pUCP20-*aadA34*	2	2	4	2	0.5	0.25	0.25	0.25	2	0.25	1	512	512

KAN, kanamycin; NEO, neomycin; PAR, paromomycin; RIB, ribostamycin; TOB, tobramycin; SIS, sisomicin; NET, netilmicin; MCR, micronomicin; SPE, spectinomycin; APR, apramycin; GEN, gentamicin; AMK, amikacin; STR, streptomycin.

The drug resistance phenotype of *aadA34* was consistent with those of other *aadA* genes. It has been reported that *aadA* genes such as *aadA16* and *aadA31* have specific resistance activity against spectinomycin and streptomycin but that they have no resistance activity to other aminoglycoside antibiotics (Cameron et al., 2018; Pinto-Alphandary et al., 1990; Clark et al., 1999; Stern et al., 2018). These results demonstrated that *aadA34* is a functional gene conferring the resistance phenotype of an *aadA* gene.

### Molecular characteristics of the novel aminoglycoside adenylyltransferase AadA34

**Figure 2:**
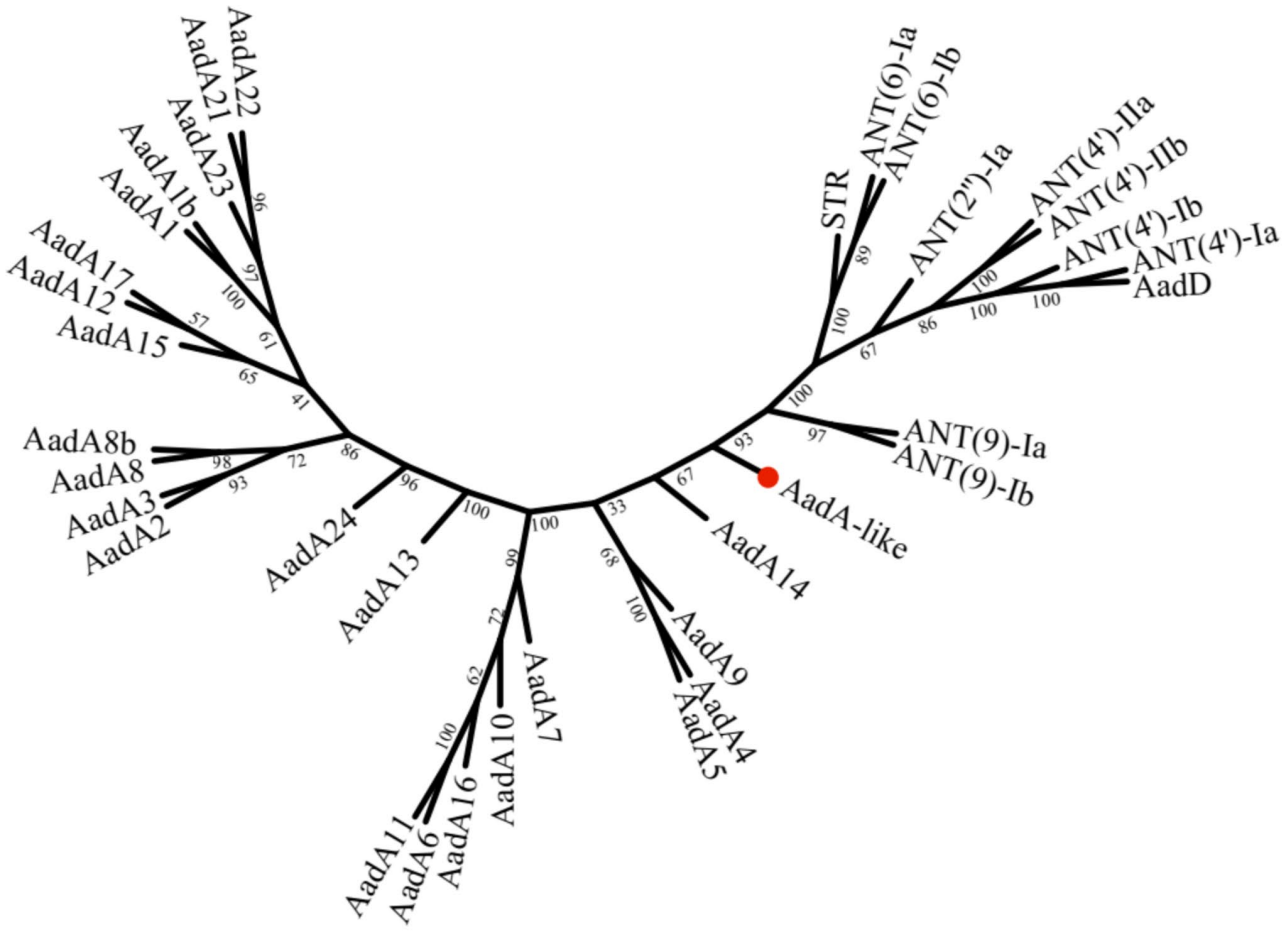
Phylogenetic analysis of AadA34 and all other functionally characterized AadAs (ANTs). The bootstrap values are shown at the nodes of the tree. AadA34 from this study is highlighted with a red filled circle. The GenBank accession numbers of the 35 proteins are as follows: AadA34, OQ862328; AadA23, CAH10847; AadA4, AAN34365; AadA12, ACJ47200; AadA9, ABG49324; AadA17, ACK43806; AadA21, AAN87151; AadA, AAO49597; AadA15, ABD58917; AadA2, AAF27727; AadA1b, AXY03807; AadA10, CAJ32491; AadA22, CAK12750; AadA5, AAF17880; AadA7, BAD00739; AadA3, AAC14728; AadA24, ABG72894; AadA16, ACF17980; AadA11, AAV32840; AadA14, CAI57696; AadA6, CAJ32504; AadA8, AAN41439; AadA13, ABW91178; AadA27, CTQ57092; AadA8b, CAJ13568; ANT(9)-Ia, CAA26963; ANT(9)-Ib, AAA16527; ANT(4′)-IIa, AAA25717; ANT(4′)-IIb, AAM76670; ANT(4′)-Ia, AAO83986; ANT(4′)-Ib, ADA62098; ANT(2″)-Ia, AAC64365; ANT(6)-Ib, CBH51824; ANT(6)-Ia, AHE40557.

The novel aminoglycoside adenylyltransferase gene *aadA34* was 1,284 bp in length and encoded a 427-aa protein, with a predicted molecular value of 47.05 kDa and an isoelectric point of 5.497. Searching AadA34 against the CARD and protein databases, among the functionally characterized proteins, AadA16 shares the highest aa similarity of approximately 46.0% (identity 56.1% and coverage 82.2%) with AadA34 and was first identified to be encoded on a plasmid of a *Vibrio cholerae* isolate from China (Sun et al., 2010). Other proteins with higher similarity with AadA34 included AadA10 (55.7%; coverage 59.3%), AadA7 (54.9%; coverage 62.2%), AadA24 (54.8%; coverage 58.1%), AadA9 (54.8%; coverage 60.1%), AadA12 (54.4%; coverage 58.3%), AadA5 (54.0%; coverage 61.4%), AadA4 (54.0%; coverage 61.2%), AadA22 (53.9%; coverage 59.1%), and AadA13 (53.6%; coverage 61.2%). The evolutionary relationships between the derived products of the *aadA* genes and other previously reported aminoglycoside adenylyltransferases were analyzed. AadA34 showed the closest evolutionary relationship to AadA14 from *Pasteurella multocida* and ANT(9)-Ic and ANT(9)-Ib from *Brucella intermedia* (Figure 2).

**Figure 3:**
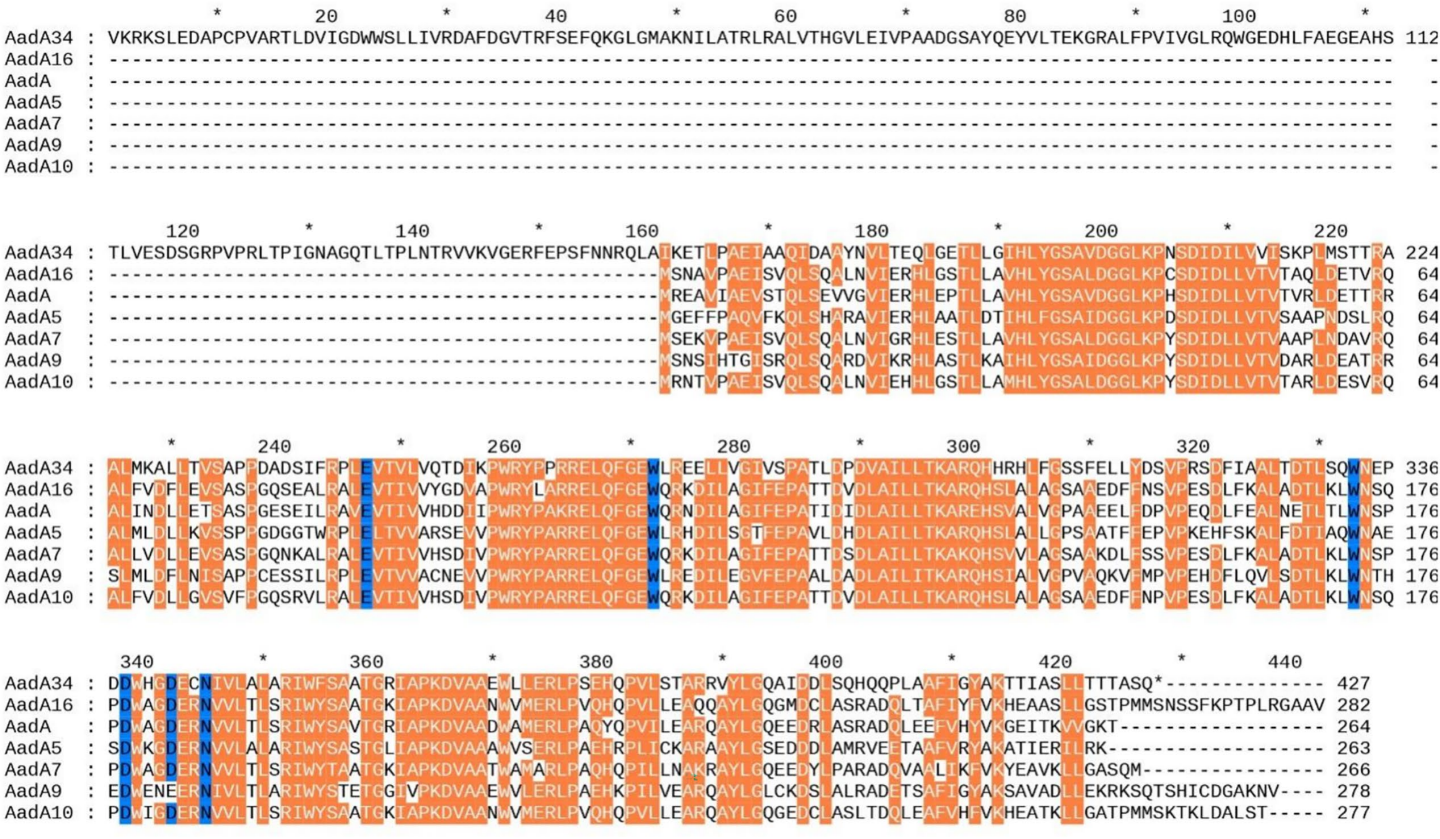
Multiple sequence alignment of amino acid sequences of AadA34 with other close relatives. Orange colored areas indicate fully conserved residues and strongly similar residues; gaps are represented using hyphens. The numbers on the right represent the corresponding sequence length. The six conserved motifs of AadA are shaded in blue. The origins of the AadAs were as follows: *Klebsiella pneumoniae* AadA16 (GenBank accession number: ACF17980), *Escherichia coli* AadA (GenBank accession number: AXY03807), *Escherichia coli* AadA5 (GenBank accession number: AAF17880), *Vibrio cholerae* AadA7 (GenBank accession number: BAD00739) and *Pseudomonas aeruginosa* AadA10 (GenBank accession number: CAJ32491), *Arthrobacter arilaitensis* AadA9 (GenBank accession number: ABG49324). The alignment was performed with MEGA12 (www.megasoftware.net).

To gain insight into the structural characteristics of the essential functional domains of AadA34, multiple sequence alignment of AadA34 with other AadA proteins, including structurally characterized AadA proteins, was performed. AadA34 had the same function-essential aa residues as the other functionally characterized aminoglycoside nucleotidyltransferases (Figure 3). Two residues (W173 and D178) and four other residues (E87, W112, D182, and N185) of AadA were reported to be responsible for the adenylation of streptomycin and spectinomycin, respectively (Stern et al., 2018), and the corresponding amino acid residues conferring the adenylation of streptomycin (W333 and D338) and spectinomycin (E247, W272, D342 and N345) were conserved in AadA34 in this work (Figure 3). These findings further confirmed that the novel aminoglycoside resistance-related protein in this study is a member of the AadA family.

Structural analysis of AadA34 demonstrated that compared with all the functionally characterized ANTs, AadA34 contained an extra fragment of approximately 160 amino acids in its N-terminus (Figure 3), which was determined to be a DNA-binding transcriptional regulator of the HxlR family (Yurimoto et al., 2005) and was generally upstream of the function-characterized ANTs previously reported. These findings revealed that the AadA34 protein might be the result of a mutation of the transcriptional regulator that led to a fusion protein composed of a transcriptional regulator and an ANT. Further analysis of the distribution of AadA34 homologous genes revealed that 73 genes with high similarity (coverage ≥97.0% and identity ≥92.7%) were found in many bacterial genomes of the genus *Serratia*, especially those of the species *Serratia marcescens* and *Serratia ureilytica*, which were isolated from various sources including many of clinical significance. Of the 27 AadA34 homologous genes analyzed, one third were from human beings (Supplementary Table S4).

### Kinetic parameters of the novel aminoglycoside nucleotidyltransferase AadA34

To characterize the enzymatic activity of AadA34, the *in vitro* kinetic parameters of AadA34 against four aminoglycosides were determined. The catalytic effects of spectinomycin and streptomycin were generally consistent with the MIC results. AadA34 showed high affinity for spectinomycin (14.5 ± 1.7 μM) and streptomycin (5.7 ± 0.9 μM). Moreover, AadA34 had a lower *k*_cat_/*K*_m_ value for spectinomycin (0.348 ± 0.051 M^−1^ s^−1^) than for streptomycin (0.482 ± 0.092 M^−1^ s^−1^) (Table 4), which indicated a weaker catalytic efficiency and lower turnover rate against spectinomycin. This phenomenon has also been reported in previous research, which was ascribed to the better structural fit of this enzyme to streptomycin (Chen et al., 2015). These results demonstrated that the catalytic activity spectrum of AadA34 was the same as those of the other AadA proteins characterized previously (Cameron et al., 2018), even though AadA34 adenylates streptomycin and spectinomycin with different catalytic efficiencies. These findings further revealed that AadA34 is a streptomycin and spectinomycin nucleotidyltransferase. No catalytic activity of AadA34 against gentamicin or kanamycin was observed.

**Table 4:** Kinetic parameters of AadA34.

Aminoglycoside	*k*_cat_ (s^−1^)^a^	*K*_m_ (μM)^a^	*k*_cat_/*K*_m_ (M^−1^ s^−1^)
Kanamycin	N^b^	N^b^	N^d^
Gentamicin	N^b^	N^b^	N^d^
Spectinomycin	5.04 ± 0.17	14.5 ± 1.7	0.348 ± 0.051
Streptomycin	2.75 ± 0.10	5.7 ± 0.9	0.482 ± 0.092

a *k*_cat_ and *K*_m_ values represent the mean ± standard deviation (SD) of three independent experiments.

b N, no enzymatic activity observed.

### The genetic context of the *aadA34* gene and its relatives

**Figure 4:**
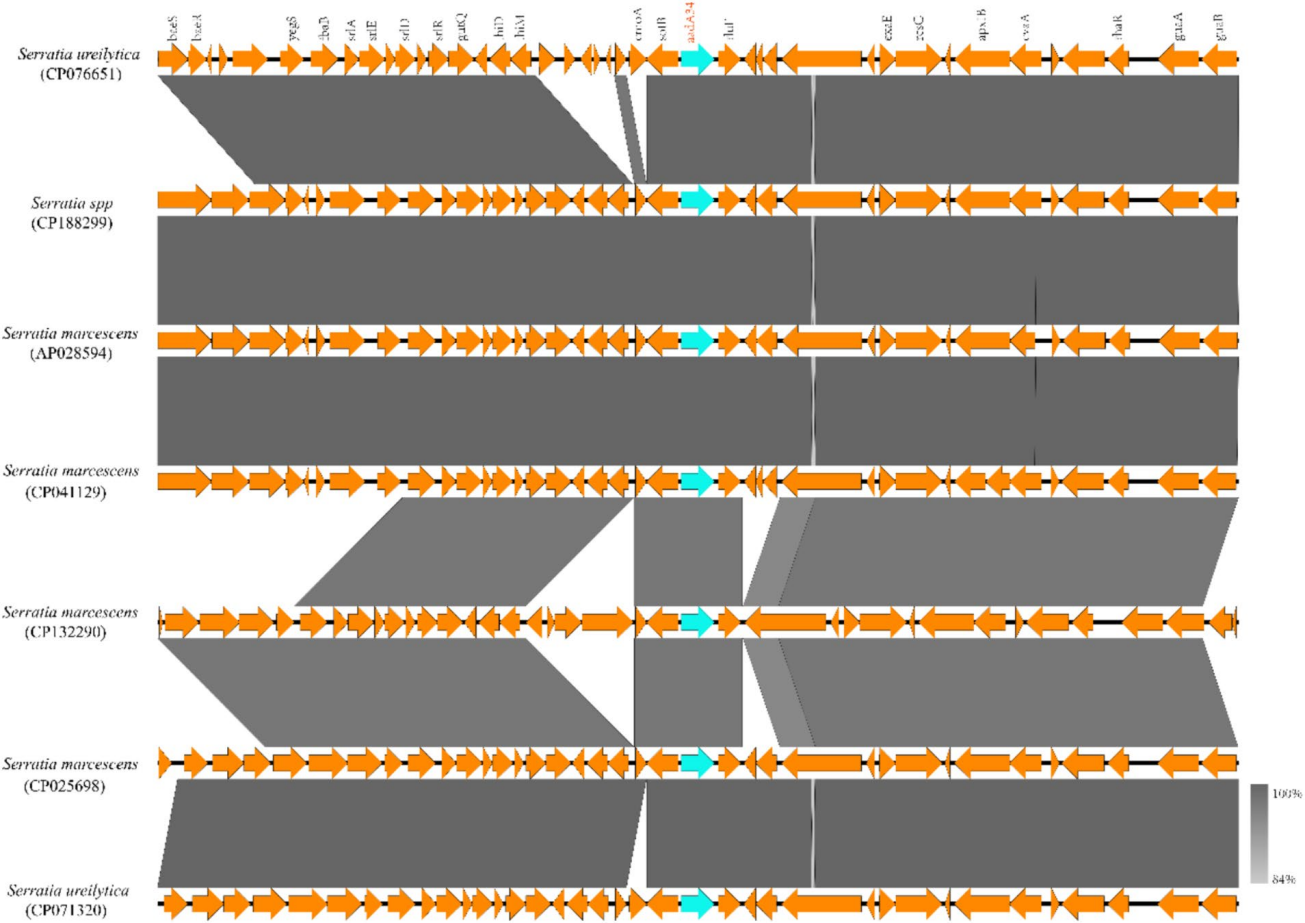
The genetic context of the *aadA34*(-like) genes. Schematic representation of the genetic environment of *aadA34*(-like) genes and comparison of the *aadA*(-like) genes carrying regions in genomes of different *Serratia* strains. ORFs are shown as arrows drawn to scale to indicate the direction of transcription. The *aadA34*(-like) genes are colored in blue and the other genes are colored in orange. The shading represent homologous regions between these genetic contexts of the sequences.

To investigate the genetic context of the aadA34-encoding region, sequences with *aadA34*(-like) genes (mentioned above) at the center of genome regions approximately 20 kb in length were retrieved from the NCBI nonredundant nucleotide database. Structural analysis revealed that, with slight differences, these sequences were generally conserved in terms of gene order and gene content (Figure 4). The genes encoding the functionally critical proteins *rluF* [a pseudouridine modification enzyme (Addepalli and Limbach, 2016)] and *sotB* [a multidrug transporter of the major facilitator superfamily (Zhai et al., 2022)] were present next to the *aadA34*(-like) genes (Figure 4). No mobile genetic elements were identified within these sequences.

## Data Availability

The datasets presented in this study can be found in online repositories. The names of the repository/repositories and accession number(s) can be found in the article/Supplementary material.

## Footnotes

1
http://web.expasy.org/docs/swiss-prot_guideline.html

2
https://card.mcmaster.ca/

3
http://itol.embl.de/

4
http://meme-suite.org/

## References

[ref1] Addepalli B. Limbach P. A. (2016). Pseudouridine in the anticodon of *Escherichia coli* tRNATyr(QPsiA) is catalyzed by the dual specificity enzyme RluF. J. Biol. Chem. 291, 22327–22337. doi: 10.1074/jbc.M116.747865, 27551044 PMC5064010

[ref2] Alcock B. P. Huynh W. Chalil R. Smith K. W. Raphenya A. R. Wlodarski M. A. . (2023). CARD 2023: expanded curation, support for machine learning, and resistome prediction at the Comprehensive Antibiotic Resistance Database. Nucleic Acids Res. 51, D690–D699. doi: 10.1093/nar/gkac920, 36263822 PMC9825576

[ref3] Brooke J. S. (2012). *Stenotrophomonas maltophilia*: an emerging global opportunistic pathogen. Clin. Microbiol. Rev. 25, 2–41. doi: 10.1128/CMR.00019-11, 22232370 PMC3255966

[ref4] Butaye P. van Duijkeren E. Prescott J. F. Schwarz S. (2014). Antimicrobial resistance in bacteria from animals and the environment. Vet. Microbiol. 171, 269–272. doi: 10.1016/j.vetmic.2014.04.009, 24852141

[ref5] Cameron A. Klima C. L. Ha R. Gruninger R. J. Zaheer R. McAllister T. A. (2018). A novel aadA aminoglycoside resistance gene in bovine and porcine pathogens. mSphere 3:e00568-17. doi: 10.1128/mSphere.00568-17, 29507894 PMC5830473

[ref6] Chen Y. Nasvall J. Wu S. Andersson D. I. Selmer M. (2015). Structure of AadA from *Salmonella enterica*: a monomeric aminoglycoside (3″)(9) adenyltransferase. Acta Crystallogr. D Biol. Crystallogr. 71, 2267–2277. doi: 10.1107/S1399004715016429, 26527143 PMC4631478

[ref7] Clark N. C. Olsvik O. Swenson J. M. Spiegel C. A. Tenover F. C. (1999). Detection of a streptomycin/spectinomycin adenylyltransferase gene (aadA) in *Enterococcus faecalis*. Antimicrob. Agents Chemother. 43, 157–160. doi: 10.1128/AAC.43.1.157, 9869582 PMC89037

[ref8] Clarridge J. E. 3rd (2004). Impact of 16S rRNA gene sequence analysis for identification of bacteria on clinical microbiology and infectious diseases. Clin. Microbiol. Rev. 17, 840–862. doi: 10.1128/CMR.17.4.840-862.2004 15489351 PMC523561

[ref9] Cortes-Rios J. Zarate A. M. Figueroa J. D. Medina J. Fuentes-Lemus E. Rodriguez-Fernandez M. . (2020). Protein quantification by bicinchoninic acid (BCA) assay follows complex kinetics and can be performed at short incubation times. Anal. Biochem. 608:113904. doi: 10.1016/j.ab.2020.113904, 32800701

[ref10] Dahdouh E. Lazaro-Perona F. Ruiz-Carrascoso G. Sanchez Garcia L. Saenz de Pipaon M. Mingorance J. (2021). Intestinal dominance by *Serratia marcescens* and *Serratia ureilytica* among neonates in the setting of an outbreak. Microorganisms 9:2271. doi: 10.3390/microorganisms9112271, 34835397 PMC8624583

[ref11] Feng C. Gao M. Jiang W. Shi W. Li A. Liu S. . (2022). Identification of a novel aminoglycoside O-nucleotidyltransferase AadA33 in *Providencia vermicola*. Front. Microbiol. 13:990739. doi: 10.3389/fmicb.2022.990739, 36177473 PMC9513248

[ref12] Florensa A. F. Kaas R. S. Clausen P. Aytan-Aktug D. Aarestrup F. M. (2022). ResFinder—an open online resource for identification of antimicrobial resistance genes in next-generation sequencing data and prediction of phenotypes from genotypes. Microb. Genom. 8:000748. doi: 10.1099/mgen.0.000748 35072601 PMC8914360

[ref13] Franklin A. M. Weller D. L. Durso L. M. Bagley M. Davis B. C. Frye J. G. . (2024). A one health approach for monitoring antimicrobial resistance: developing a national freshwater pilot effort. Front. Water 6:1359109. doi: 10.3389/frwa.2024.1359109, 38855419 PMC11157689

[ref14] Gomi R. Matsumura Y. Tanaka M. Ihara M. Sugie Y. Matsuda T. . (2022). Emergence of rare carbapenemases (FRI, GES-5, IMI, SFC and SFH-1) in Enterobacterales isolated from surface waters in Japan. J. Antimicrob. Chemother. 77, 1237–1246. doi: 10.1093/jac/dkac029, 35137119

[ref15] Grin I. Linke D. (2011). GCView: the genomic context viewer for protein homology searches. Nucleic Acids Res. 39, W353–W356. doi: 10.1093/nar/gkr364 21609955 PMC3125770

[ref16] Heuer O. E. Kruse H. Grave K. Collignon P. Karunasagar I. Angulo F. J. (2009). Human health consequences of use of antimicrobial agents in aquaculture. Clin. Infect. Dis. 49, 1248–1253. doi: 10.1086/605667, 19772389

[ref17] Holloway W. J. (1982). Spectinomycin. Med. Clin. North Am. 66, 169–173. doi: 10.1016/S0025-7125(16)31450-X, 6460907

[ref18] Hopkins K. L. Findlay J. Meunier D. Cummins M. Curtis S. Kustos I. . (2017). *Serratia marcescens* producing SME carbapenemases: an emerging resistance problem in the UK? J. Antimicrob. Chemother. 72, 1535–1537. doi: 10.1093/jac/dkw567, 28108680

[ref19] Jo H. (2016). Multi-threading the generation of Burrows–Wheeler Alignment. Genet. Mol. Res. 15:gmr.15028650. doi: 10.4238/gmr.15028650, 27323088

[ref20] Kim C. Hesek D. Zajicek J. Vakulenko S. B. Mobashery S. (2006). Characterization of the bifunctional aminoglycoside-modifying enzyme ANT(3″)-Ii/AAC(6′)-IId from *Serratia marcescens*. Biochemistry 45, 8368–8377. doi: 10.1021/bi060723g, 16819836

[ref21] Kumar S. Stecher G. Li M. Knyaz C. Tamura K. (2018). MEGA X: molecular evolutionary genetics analysis across computing platforms. Mol. Biol. Evol. 35, 1547–1549. doi: 10.1093/molbev/msy096, 29722887 PMC5967553

[ref22] Lawton J. G. Zhou A. E. Stucke E. M. Takala-Harrison S. Silva J. C. Travassos M. A. (2025). Diamonds in the rif: alignment-free comparative genomics analysis reveals strain-transcendent plasmodium falciparum antigens amidst extensive genetic diversity. Infect. Genet. Evol. 129:105725. doi: 10.1016/j.meegid.2025.105725, 39920908 PMC12032969

[ref23] Li J. Han Q. Zhang T. Du J. Sun Q. Pang Y. (2018). Expression of soluble native protein in *Escherichia coli* using a cold-shock SUMO tag-fused expression vector. Biotechnol. Rep. 19:e00261. doi: 10.1016/j.btre.2018.e00261, 30009138 PMC6042314

[ref24] Lu W. Li K. Huang J. Sun Z. Li A. Liu H. . (2021). Identification and characteristics of a novel aminoglycoside phosphotransferase, APH(3′)-IId, from an MDR clinical isolate of *Brucella intermedia*. J. Antimicrob. Chemother. 76, 2787–2794. doi: 10.1093/jac/dkab272, 34329431

[ref25] Mahlen S. D. (2011). *Serratia* infections: from military experiments to current practice. Clin. Microbiol. Rev. 24, 755–791. doi: 10.1128/CMR.00017-11, 21976608 PMC3194826

[ref26] Manaia C. M. (2017). Assessing the risk of antibiotic resistance transmission from the environment to humans: non-direct proportionality between abundance and risk. Trends Microbiol. 25, 173–181. doi: 10.1016/j.tim.2016.11.014, 28012687

[ref27] McKenna A. Hanna M. Banks E. Sivachenko A. Cibulskis K. Kernytsky A. . (2010). The genome analysis toolkit: a MapReduce framework for analyzing next-generation DNA sequencing data. Genome Res. 20, 1297–1303. doi: 10.1101/gr.107524.110, 20644199 PMC2928508

[ref28] Messaoudi A. Mansour W. Tilouche L. Chatre P. Drapeau A. Chaouch C. . (2021). First report of carbapenemase OXA-181-producing *Serratia marcescens*. J. Glob. Antimicrob. Resist. 26, 205–206. doi: 10.1016/j.jgar.2021.06.004, 34242801

[ref29] Mischnik A. Baumert P. Hamprecht A. Rohde A. M. Peter S. Feihl S. . (2017). In vitro susceptibility to 19 agents other than beta-lactams among third-generation cephalosporin-resistant Enterobacteriaceae recovered on hospital admission. J. Antimicrob. Chemother. 72, 1359–1363. doi: 10.1093/jac/dkw577, 28108677

[ref30] Mitteer D. R. Greer B. D. (2022). Using GraphPad prism’s heat maps for efficient, fine-grained analyses of single-case data. Behav. Anal. Pract. 15, 505–514. doi: 10.1007/s40617-021-00664-7, 35692516 PMC9120324

[ref31] Mukherjee N. Nolan V. G. Dunn J. R. Banerjee P. (2019). Sources of human infection by *Salmonella enterica* serotype Javiana: a systematic review. PLoS One 14:e0222108. doi: 10.1371/journal.pone.0222108, 31479476 PMC6719869

[ref32] Nguyen H. N. Van T. T. Nguyen H. T. Smooker P. M. Shimeta J. Coloe P. J. (2014). Molecular characterization of antibiotic resistance in *Pseudomonas* and *Aeromonas* isolates from catfish of the Mekong Delta, Vietnam. Vet. Microbiol. 171, 397–405. doi: 10.1016/j.vetmic.2014.01.028, 24629778

[ref33] Nordmann P. Poirel L. (2019). Epidemiology and diagnostics of carbapenem resistance in Gram-negative bacteria. Clin. Infect. Dis. 69, S521–S528. doi: 10.1093/cid/ciz824, 31724045 PMC6853758

[ref34] Pinto-Alphandary H. Mabilat C. Courvalin P. (1990). Emergence of aminoglycoside resistance genes aadA and aadE in the genus Campylobacter. Antimicrob. Agents Chemother. 34, 1294–1296. doi: 10.1128/AAC.34.6.1294, 2168151 PMC171807

[ref35] Rhouma M. Beaudry F. Letellier A. (2016). Resistance to colistin: what is the fate for this antibiotic in pig production? Int. J. Antimicrob. Agents 48, 119–126. doi: 10.1016/j.ijantimicag.2016.04.008, 27234675

[ref36] Schatz A. Bugie E. Waksman S. A. (2005). Streptomycin, a substance exhibiting antibiotic activity against gram-positive and gram-negative bacteria. 1944. Clin. Orthop. Relat. Res. 437, 3–6. doi: 10.1097/01.blo.0000175887.98112.fe 16056018

[ref37] Seemann T. (2014). Prokka: rapid prokaryotic genome annotation. Bioinformatics 30, 2068–2069. doi: 10.1093/bioinformatics/btu153, 24642063

[ref38] Shaw K. J. Rather P. N. Sabatelli F. J. Mann P. Munayyer H. Mierzwa R. . (1992). Characterization of the chromosomal aac(6′)-Ic gene from *Serratia marcescens*. Antimicrob. Agents Chemother. 36, 1447–1455. doi: 10.1128/AAC.36.7.1447, 1354954 PMC191602

[ref39] Stern A. L. Van der Verren S. E. Kanchugal P. S. Nasvall J. Gutierrez-de-Teran H. Selmer M. (2018). Structural mechanism of AadA, a dual-specificity aminoglycoside adenylyltransferase from *Salmonella enterica*. J. Biol. Chem. 293, 11481–11490. doi: 10.1074/jbc.RA118.003989, 29871922 PMC6065190

[ref40] Sullivan M. J. Petty N. K. Beatson S. A. (2011). Easyfig: a genome comparison visualizer. Bioinformatics 27, 1009–1010. doi: 10.1093/bioinformatics/btr039, 21278367 PMC3065679

[ref41] Sun J. Zhou M. Wu Q. Ni Y. (2010). Characterization of two novel gene cassettes, dfrA27 and aadA16, in a non-O1, non-O139 *Vibrio cholerae* isolate from China. Clin. Microbiol. Infect. 16, 1125–1129. doi: 10.1111/j.1469-0691.2009.03060.x, 19906273

[ref42] Theuretzbacher U. Van Bambeke F. Canton R. Giske C. G. Mouton J. W. Nation R. L. . (2015). Reviving old antibiotics. J. Antimicrob. Chemother. 70, 2177–2181. doi: 10.1093/jac/dkv157 26063727

[ref43] Thompson C. C. Chimetto L. Edwards R. A. Swings J. Stackebrandt E. Thompson F. L. (2013). Microbial genomic taxonomy. BMC Genomics 14:913. doi: 10.1186/1471-2164-14-913, 24365132 PMC3879651

[ref44] Tolmasky M. E. (1990). Sequencing and expression of aadA, Bla, and tnpR from the multiresistance transposon Tn1331. Plasmid 24, 218–226. doi: 10.1016/0147-619X(90)90005-W, 1963948

[ref45] Versmessen N. Mispelaere M. Vandekerckhove M. Hermans C. Boelens J. Vranckx K. . (2024). Average nucleotide identity and digital DNA-DNA hybridization analysis following PromethION nanopore-based whole genome sequencing allows for accurate prokaryotic typing. Diagnostics 14:1800. doi: 10.3390/diagnostics14161800, 39202288 PMC11353866

[ref46] Wiegand I. Hilpert K. Hancock R. E. (2008). Agar and broth dilution methods to determine the minimal inhibitory concentration (MIC) of antimicrobial substances. Nat. Protoc. 3, 163–175. doi: 10.1038/nprot.2007.521, 18274517

[ref47] Wingett S. W. Andrews S. (2018). FastQ screen: a tool for multi-genome mapping and quality control. F1000Res 7:1338. doi: 10.12688/f1000research.15931.1, 30254741 PMC6124377

[ref48] Yurimoto H. Hirai R. Matsuno N. Yasueda H. Kato N. Sakai Y. (2005). HxlR, a member of the DUF24 protein family, is a DNA-binding protein that acts as a positive regulator of the formaldehyde-inducible hxlAB operon in *Bacillus subtilis*. Mol. Microbiol. 57, 511–519. doi: 10.1111/j.1365-2958.2005.04702.x, 15978081

[ref49] Zhai G. Zhang Z. Dong C. (2022). Mutagenesis and functional analysis of SotB: a multidrug transporter of the major facilitator superfamily from *Escherichia coli*. Front. Microbiol. 13:1024639. doi: 10.3389/fmicb.2022.1024639, 36386622 PMC9650428

