# Supplementary material for: Characterization of a novel aminoglycoside resistance gene, aadA34, identified from Serratia ureilytica S24
Source: Front Microbiol. 2026 Mar 18;17:1765554. doi: 10.3389/fmicb.2026.1765554 (PMC13038893; doi:10.3389/fmicb.2026.1765554)
Supplement: Supplementary file 1 [file Table_1.DOCX]

**Table S1. Bacteria and plasmids used in this work.**

| Strain and plasmid | Description | Source |
| --- | --- | --- |
| S24 | The wild-type strain of *S. ureilytica* S24 | This work |
| BL21 | *Escherichia coli* BL21 as a host for expression of the AadA34 protein | Our laboratory collection |
| DH5α | *Escherichia coli* DH5α as a host for cloning of the aadA34 gene | Our laboratory collection |
| ATCC25922 | *Escherichia coli* ATCC 25922 as a quality control for antimicrobial susceptibility testing | Our laboratory collection |
| ATCC27853 | Pseudomonas *aruginosa* ATCC 27853 as quality control for antimicrobial susceptibility testing | Our laboratory collection |
| pUCP20 | Cloning vector for the PCR products of the *aadA34* gene with its upstream promoter region, AMP^r^ | Our laboratory collection |
| pCold1 | Expression vector for the PCR products of the ORF of the *aadA34* gene, AMP^r^ | Our laboratory collection |
| pUCP20-*aadA34*/DH5α | DH5α carrying the recombinant plasmid pUCP20-*aadA34* | This work |
| pCold 1-*aadA34*/ BL21 | BL21 carrying the recombinant plasmid pCold I-*aadA34* | This work |

^r^ resistance.

**Table S2. Antibiotics resistance phenotype of *S. ureilytica* S24 (μg/mL).**

| **antibiotics** | **S24** | **antibiotics** | **S24** | **antibiotics** | **S24** |
| --- | --- | --- | --- | --- | --- |
| Chloramphenicol | 8^a^ (S^d^) | Tetracycline | 32^a^ (R^b^) | Kanamycin | 2^a^ (S^d^) |
| Florfenicol | 8^a^ (S^d^) | Tigecycline | 0.5^a^ (S^d^) | Paromomycin | 16^a^ (I^c^) |
| Ciprofloxacin | 64^a^ (R^b^) | Minocycline | 1^a^ (S^d^) | Spectinomycin | 64^a^ (R^b^) |
| Enrofloxacin | 32^a^ (R^b^) | Ceftazidime | 0.5^a^ (S^d^) | Streptomycin | 32^a^ (R^b^) |
| Levofloxacin | 32^a^ (R^b^) | Ceftiofur | 64^a^ (R^b^) | Neomycin | 2^a^ (S^d^) |
| Fosfomycin | 128^a^ (R^b^) | Aztreonam | 8^a^ (S^d^) | Sisomicin | 32^a^ (R^b^) |
| Polymycin B | 8^a^ (S^d^) | Ampicillin | >512^a^ (R^b^) | Ribostamycin | 64^a^ (R^b^) |
| Gentamicin | 0.25^a^ (S^d^) | Imipenem | 0.125^a^ (S^d^) | Tobramycin | 64^a^ (R^b^) |
| Amikacin | 4^a^ (I^c^) | Trimethoprim | 16^a^ (I^c^) | Micronomicin | 64^a^ (R^b^) |

^a^ the antimicrobials with CLSI, ECAST or NARMS resistance breakpoint criteria for enteric bacteria; ^b^ resistance; ^c^ intermedium; ^d^ sensitive.

**Table S3. The putative resistance genes predicted in the isolate *S. ureilytica* S24 genome.**

| **Putative resistance genes** | **Length (bp)** | **Identity (%)** | **GenPept accession no. of the closest homologue** |
| --- | --- | --- | --- |
| *aadA16-like* | 1,284 | 56.06 | EU675686.2 [*aadA16*] |
| *aph(6)Ic-like* | 1,056 | 49.06 | X01702.1 [*aph(6)-Ic*] |
| *aac(3)Ib-like* | 720 | 47.62 | NG_067945 [*aac(3)-Ib*] |
| *aac(6')Ib-like* | 630 | 38.01 | M55547.1 [*aac(6')-Ib*] |
| *aac(6')Iy-like* | 675 | 37.02 | AF144881 [*aac(6')-Iy*] |
| *aac(6')Iak-like* | 528 | 33.06 | AB894482 [*aac(6')-Iak*] |

**Table S4. Similar genes of *aadA34* found in the NCBI nucleotide database.**

| Description | Scientific Name | Query Cover | Identy | Accession NO. | Source |  |
| --- | --- | --- | --- | --- | --- | --- |
| hypothetical protein SMKC081_30030 [Serratia marcescens] | Serratia marcescens | 100% | 100 | BEN74858.1 | this work |  |
| adenylyltransferase [Serratia marcescens] | Serratia marcescens | 100% | 99.77 | OPJ91962.1 | Factory soil | |
| DUF4111 domain-containing protein [Serratia marcescens] | Serratia marcescens | 100% | 99.77 | EMF6290107.1 | farm soil |  |
| DUF4111 domain-containing protein [Serratia marcescens] | Serratia marcescens | 100% | 99.53 | EMD1304612.1 | Homo sapiens | |
| DUF4111 domain-containing protein [Serratia ureilytica] | Serratia ureilytica | 100% | 99.53 | MBL0877957.1 | veterinary hospital | |
| DUF4111 domain-containing protein [Serratia ureilytica] | Serratia ureilytica | 100% | 99.53 | MBH3267655.1 | soil |  |
| hypothetical protein SME10J_30470 [Serratia marcescens] | Serratia marcescens | 100% | 99.53 | BEM39320.1 | water |  |
| Streptomycin 3''-adenylyltransferase, partial [Serratia marcescens] | Serratia marcescens | 100% | 99.77 | CAH5408778.1 | Homo sapiens | |
| DUF4111 domain-containing protein [Serratia ureilytica] | Serratia ureilytica | 100% | 99.3 | MBS7521359.1 | soil |  |
| DUF4111 domain-containing protein [Serratia ureilytica] | Serratia ureilytica | 100% | 99.53 | MDM1843714.1 | Homo sapiens | |
| DUF4111 domain-containing protein [Serratia marcescens] | Serratia marcescens | 100% | 99.53 | EMF7441251.1 | soil |  |
| hypothetical protein SME17J_30410 [Serratia marcescens] | Serratia marcescens | 100% | 99.53 | BEM49547.1 | Homo sapiens | |
| aminoglycoside nucleotidyltransferase [Serratia marcescens] | Serratia marcescens | 100% | 99.53 | AWO80383.1 | Homo sapiens | |
| DUF4111 domain-containing protein [Serratia marcescens] | Serratia marcescens | 100% | 99.53 | AUO04984.1 | Solanacea rhizosphere | |
| Streptomycin 3''-adenylyltransferase [Serratia marcescens] | Serratia marcescens | 100% | 99.3 | CAI2510119.1 | Bos taurus | |
| DUF4111 domain-containing protein [Serratia marcescens] | Serratia marcescens | 100% | 99.3 | EKX2169019.1 | soil |  |
| Aminoglycoside nucleotidyltransferase [Serratia marcescens] | Serratia marcescens | 100% | 99.3 | AXK25512.1 | Bos taurus | |
| DUF4111 domain-containing protein [Serratia marcescens] | Serratia marcescens | 100% | 99.3 | MBN5292654.1 | freshwater: disused tungsten mine | |
| DUF4111 domain-containing protein [Serratia marcescens] | Serratia marcescens | 100% | 99.06 | UIM54411.1 | soil |  |
| DUF4111 domain-containing protein [Serratia marcescens] | Serratia marcescens | 100% | 99.3 | HEJ9081005.1 | soil |  |
| DUF4111 domain-containing protein [Serratia ureilytica] | Serratia ureilytica | 100% | 99.3 | MBF8440822.1 | Homo sapiens | |
| DUF4111 domain-containing protein [Serratia ureilytica] | Serratia ureilytica | 100% | 99.3 | MBO1808824.1 | Bos taurus | |
| DUF4111 domain-containing protein [Serratia marcescens] | Serratia marcescens | 100% | 99.06 | HEJ8017672.1 | Solanum nigrum | |
| aminoglycoside adenylyltransferase family protein [Serratia marcescens] | Serratia marcescens | 100% | 99.53 | MDP0521597.1 | Homo sapiens | |
| DUF4111 domain-containing protein [Serratia ureilytica] | Serratia ureilytica | 100% | 99.06 | MBH2802346.1 | water |  |
| DUF4111 domain-containing protein [Serratia ureilytica] | Serratia ureilytica | 100% | 98.83 | MBJ2090732.1 | Homo sapiens | |
| adenylyltransferase [Serratia marcescens] | Serratia marcescens | 100% | 98.83 | PHY80721.1 | Homo sapiens | |
| hypothetical protein AF54_02441 [Serratia marcescens BIDMC 81] | Serratia marcescens BIDMC 81 | 100% | 98.59 | EZQ61776.1 | soil |  |
| DUF4111 domain-containing protein [Serratia ureilytica] | Serratia ureilytica | 100% | 98.83 | MBJ2105814.1 | soil |  |
